# Usability Testing and Technology Acceptance of an mHealth App at the Point of Care During Simulated Pediatric In- and Out-of-Hospital Cardiopulmonary Resuscitations: Study Nested Within 2 Multicenter Randomized Controlled Trials

**DOI:** 10.2196/35399

**Published:** 2022-03-01

**Authors:** Johan N Siebert, Laëtitia Gosetto, Manon Sauvage, Laurie Bloudeau, Laurent Suppan, Frédérique Rodieux, Kevin Haddad, Florence Hugon, Alain Gervaix, Christian Lovis, Christophe Combescure, Sergio Manzano, Frederic Ehrler, PedAMINES Trial Group, PedAMINES Prehospital Group

**Affiliations:** 1 Department of Pediatric Emergency Medicine Geneva Children’s Hospital Geneva University Hospitals Geneva Switzerland; 2 Faculty of Medicine University of Geneva Geneva Switzerland; 3 Diagnostic Department Geneva University Hospitals Geneva Switzerland; 4 A.C.E. Geneva Ambulances SA Geneva Switzerland; 5 Department of Emergency Medicine Geneva University Hospitals Geneva Switzerland; 6 Division of Clinical Pharmacology and Toxicology Department of Anesthesiology, Clinical Pharmacology, Intensive Care and Emergency Medicine Geneva University Hospitals Geneva Switzerland; 7 Division of Medical Information Sciences Department of Radiology and Medical Informatics Geneva University Hospitals Geneva Switzerland; 8 Division of Clinical Epidemiology Department of Health and Community Medicine Geneva University Hospitals Geneva Switzerland; 9 See Acknowledgments

**Keywords:** cardiopulmonary resuscitation, drugs, emergency medical services, medication errors, mobile health, mobile apps, out-of-hospital cardiac arrest, paramedics, pediatrics, System Usability Scale, Unified Theory of Acceptance and Use of Technology, smartphone, mobile phone

## Abstract

**Background:**

Mobile apps are increasingly being used in various domains of medicine. Few are evidence-based, and their benefits can only be achieved if end users intend to adopt and use them. To date, only a small fraction of mobile apps have published data on their field usability and end user acceptance results, especially in emergency medicine.

**Objective:**

This study aims to determine the usability and acceptance of an evidence-based mobile app while safely preparing emergency drugs at the point of care during pediatric in- and out-of-hospital cardiopulmonary resuscitations by frontline caregivers.

**Methods:**

In 2 multicenter randomized controlled parent trials conducted at 6 pediatric emergency departments from March 1 to December 31, 2017, and 14 emergency medical services from September 3, 2019, to January 21, 2020, the usability and technology acceptance of the PedAMINES (Pediatric Accurate Medication in Emergency Situations) app were evaluated among skilled pediatric emergency nurses and advanced paramedics when preparing continuous infusions of vasoactive drugs and direct intravenous emergency drugs at pediatric dosages during standardized, simulation-based, pediatric in- and out-of-hospital cardiac arrest scenarios, respectively. Usability was measured using the 10-item System Usability Scale. A 26-item technology acceptance self-administered survey (5-point Likert-type scales), adapted from the Unified Theory of Acceptance and Use of Technology model, was used to measure app acceptance and intention to use.

**Results:**

All 100% (128/128) of nurses (crossover trial) and 49.3% (74/150) of paramedics (parallel trial) were assigned to the mobile app. Mean total scores on the System Usability Scale were excellent and reached 89.5 (SD 8.8; 95% CI 88.0-91.1) for nurses and 89.7 (SD 8.7; 95% CI 87.7-91.7) for paramedics. Acceptance of the technology was very good and rated on average >4.5/5 for 5 of the 8 independent constructs evaluated. Only the image construct scored between 3.2 and 3.5 by both participant populations.

**Conclusions:**

The results provide evidence that dedicated mobile apps can be easy to use and highly accepted at the point of care during in- and out-of-hospital cardiopulmonary resuscitations by frontline emergency caregivers. These findings can contribute to the implementation and valorization of studies aimed at evaluating the usability and acceptance of mobile apps in the field by caregivers, even in critical situations.

**Trial Registration:**

ClinicalTrials.gov NCT03021122; https://clinicaltrials.gov/ct2/show/NCT03021122. ClinicalTrials.gov NCT03921346; https://clinicaltrials.gov/ct2/show/NCT03921346

**International Registered Report Identifier (IRRID):**

RR2-10.1186/s13063-019-3726-4

## Introduction

### Background

Over the past few decades, health information technologies (HITs) and communication technologies have been widely adopted in health care environments to improve care provision, efficiency, quality, and patient safety while achieving cost savings [1]. Supported by the rapid spread of mobile devices and their innovative features (eg, connectivity, on-board computing capabilities, small size, and operating systems allowing mobile app development), mobile health (mHealth) has undergone considerable development to address health issues by providing medical and communication services within easy reach of end users [2-4]. As of the first quarter of 2021, approximately 5.7 million apps were available on leading web-based app stores [5]. Among them, >325,000 are mHealth apps [6]. The vast majority (65%) are wellness apps designed to be used primarily by the general public. Approximately 15% are patient-centered apps and focus on self-management of specific conditions, and the remaining 20% are medical apps intended for health care providers [7]. Unfortunately, most do not adhere to relevant medical evidence and lack expert involvement in their development or validation process through high-quality studies to support their adoption in clinical practice [2,8-13]. Even when apps are evidence-based, this does not guarantee that they will be used consistently over time. As with other HIT, their benefits can only be achieved if end users intend to adopt them [10,14].

Understanding users’ HIT adoption behavior is a long-standing topic in the literature and could lead to improvements in the acceptability and use of mHealth apps. Several models have been proposed to predict and understand users’ acceptance and use of HIT. Two of the most commonly used models are the Technology Acceptance Model (TAM) [15,16], or its extended versions (TAM Task–Technology Fit [TTF] [17], TAM2 [18], and TAM3 [19]), and the Unified Theory of Acceptance and Use of Technology (UTAUT; version 1 [20] or 2 [21]). However, a systematic review identified that mobile apps were the least frequently studied HIT application areas using the TAM, with only 15 studies published between 1989 (ie, the year when the TAM was introduced) and 2017 [22]. Although the UTAUT has a better explanatory power for technology acceptance [23] and has been extensively used in studies related to the adoption of HIT, user acceptance and use of medical apps from the perspective of caregivers have been little investigated by this model [24]. In addition, even if usability has been identified as a key component of good practice in the development of digital apps [25], the number of medical apps that publish their usability evaluation results remains scarce [26]. To this end, the validated System Usability Scale (SUS) has been identified as the most frequently used questionnaire [27-29]; however, it concerns mostly mobile apps designed to support patients in health self-management and not caregivers [26]. As a result, little is known about health professionals’ willingness to implement and use medical apps in clinical care [26,30,31].

### Previous Work

In previous randomized trials, we reported fewer medication errors and a shorter time to drug preparation and delivery during out-of-hospital cardiac arrest (OHCA) and in-hospital post–cardiac arrest scenarios when using the PedAMINES (Pediatric Accurate Medication in Emergency Situations) app than conventional preparation methods [32,33]. This evidence-based app was designed as a step-by-step guide for the preparation and delivery of intravenous drugs to address the unmet need for reducing pediatric medication errors [34]. Recent findings showed that this app was also able to reduce acute perceived stress while preparing emergency drugs in a prehospital setting during pediatric OHCA in a simulated model [35]. However, its usability and technology acceptance by frontline caregivers remains to be determined.

### Aim

This study aims to investigate the usability of the PedAMINES app by both advanced paramedics with drug preparation autonomy and nurses at the point of care to gain insight into their perceptions of its adoption as an approach to facilitate emergency drug preparation in pediatric life-threatening situations. In addition, we measure the technology acceptance of the app for its intended purpose and user satisfaction with its use. We hypothesize that this approach would help estimate the likelihood of adoption of the app for implementation among its future target users.

## Methods

### Study Design

This is a nested, overlapping study within the context of 2 prospective, multicenter, randomized controlled trials registered at ClinicalTrials.gov (NCT03021122 and NCT03921346). The parent trials had the broader and primary aim of assessing rates of medication dosing errors during simulation-based pediatric OHCA and in-hospital post–cardiac arrest scenarios using a high-fidelity manikin [32,33]. For that purpose, participants were randomly assigned (1:1 ratio) to prepare the drugs either with the support of the app (intervention group) or by conventional methods (control group). The trial protocols containing details of the scenarios have been previously published [36,37]. Both trials were performed in accordance with appropriate guidelines [38,39] and followed the CONSORT (Consolidated Standards of Reporting Trials) reporting guidelines [40].

### Technology Acceptance Terminology and Definition

The terms *technology acceptability*, *acceptance*, and *adoption* are often used confusingly or interchangeably in the mHealth literature. For the purposes of this paper, the term *technology acceptance* is used and should be understood as referring to *initial acceptance of use*, as recently defined by Nadal et al [41], to explicitly distinguish between the different temporal stages of technological acceptance. It refers to users’ first interactions with the app at the preadoption stage before sustained acceptance of its use in the postadoption stage.

### Setting and Intervention

The first trial [32] was conducted at 14 emergency medical services (EMSs). An out-of-hospital child’s bedroom environment was simulated at each EMS center to resemble, as much as possible, a standard environment where paramedics would have to intervene. Participants were tested on sequential preparations of four direct intravenous emergency drugs of varying degrees of preparation difficulty (epinephrine, midazolam, 10% dextrose, and sodium bicarbonate) during simulated pediatric OHCA. The second trial [33] was conducted as a crossover study at 6 pediatric emergency departments. Participants were tested on the preparation of continuous infusions (dopamine and norepinephrine) during simulated pediatric immediate post–cardiac arrest scenarios. Procedures were standardized across all sites to follow the same chronological progression and range of difficulty to ensure that each participant was exposed to exactly the same case in their respective setting, with similar challenges in app use and decision-making. The scripted and uniform delivery of the scenarios throughout the studies was strictly preserved to minimize confounders. Importantly, we did not organize pretests to minimize preparation bias so as not to influence the usability and acceptability of the app based on previous experiences. In both trials, the app was interfaced on an Apple iPad with the latest version of iOS; however, the app works identically on the Android OS (Google Inc). In both trials, all participants who had used the app were surveyed with self-administered questionnaires (refer to the following sections) immediately after completing the scenarios, with the necessary precautions taken to ensure that participants could not communicate with each other. During completion of the questionnaires, no interaction occurred between the participants and investigators other than those related to detailing an item upon the request of a participant.

### Participants

All registered paramedics and pediatric nurses working at 14 EMSs and 6 academic and community Swiss pediatric emergency departments, respectively, were eligible for inclusion in the study. Participants were of both sexes, of all ages, of different levels of pediatric experience, and from different regions of Switzerland, a pluralistic country with several official languages (French, German, and Italian), to diversify participant characteristics and limit selection bias. Apart from language translation, no other transcultural adaptations were required or made to the app. The inclusion criteria were to have followed a standardized 5-minute introductory course on the use of the mobile device app and written informed consent. This introductory course was not intended at this stage to test the usability of the app but to explain its use for the upcoming intervention. Only participants who had used the app (ie, all those enrolled in the in-hospital study [crossover trial] and half of those enrolled in the out-of-hospital study [parallel group trial]) were eligible for this study. All participants were assumed to have equivalent experience and competence with intravenous drug preparation and dose calculation, as this is part of their regular practice and training background. Given that this study was nested within both randomized trials, the number of participants queried in each trial stemmed from the power calculations set for the primary outcome, with a 2-sided risk α of .05 and a power of 90%. Blinding to the purpose of each trial during recruitment was maintained to minimize the preparation bias. Participants were unblinded after randomization at the beginning of the scenario. Although the intervention could not be masked, all investigators remained unaware of the outcomes until all data were unlocked for analysis at the end of the trial.

### Measurement Instruments

On the day of participation after randomized allocation and before scenarios started, each participant was required to complete a survey collecting data regarding their demographic characteristics and health care training. Five-point Likert-type scales, ranging from 1=*strongly disagree* to 5=*strongly agree*, were used to assess (1) their experience in the use of smartphones and tablets, (2) satisfaction with current supports at their disposal to prepare emergency intravenous drugs, (3) perceived mastery of the preparation of these drugs, (4) propensity to use technological tools in emergency situations, and (5) attitude toward the introduction of technological tools to facilitate the preparation of intravenous drugs during an emergency. Each participant was then exposed to the simulated out-of-hospital or in-hospital scenarios.

After the scenarios were completed, the perceived usability of the app was measured using the reliable SUS designed by Brooke [27]. According to the International Organization for Standardization, the SUS assesses effectiveness (ie, the ability of users to use the product), efficiency (ie, the effort to use the product), and satisfaction (ie, how the users felt when using the product). It comprises a 10-item questionnaire with 5 response options for each item based on their level of agreement, ranging from 1 (*strongly disagree*) to 5 (*strongly agree*). According to the scoring system by Brooke, for odd-numbered (1, 3, 5, 7, and 9) statements (the positively worded items), the score contribution is equal to the scale position minus 1 (eg, *strongly agree*: 5 – 1=4). For even-numbered (2, 4, 6, 8, and 10) statements (the negatively worded items), the score contribution is equal to 5 minus the scale position (eg, *strongly agree*: 5 – 5=0). Each score contribution falls within the range of 0 to 4. The participants’ scores for each item are then added up together and multiplied by 2.5 to convert the original scores of 0 to 40 to 0 to 100. Although the scores range from 0 to 100, these are not percentages of usability. The higher the score, the better the usability (ie, 0=*very poor perceived usability* and 100=*excellent perceived usability*). To obtain an SUS score of 100, the respondent must answer 5 to all odd questions and 0 to all even questions. The original SUS items are presented in Multimedia Appendix 1 [27]. When translating the SUS questionnaire from its original version to French, German, and Italian [42,43], we replaced the general term *system* with the specific term *PedAMINES*.

Acceptability and usability testing of the app was also assessed in both trials using a tailored 26-item technology acceptance self-administered survey. Scales and queries with high levels of internal consistency (ie, Cronbach α >.70) were derived and slightly adapted from prior research to fit the trial’s context (Multimedia Appendix 2) [44]. This integrative model gathers the 2 most commonly used models in the literature that inform technology acceptance [45], namely the TAM [15] and UTAUT [20,21], as well as additional dimensions of technology acceptance models [19,20,46-50], with the following eight core constructs: (1) perceived usefulness (4 items), (2) perceived ease of use (4 items), (3) TTF (4 items), (4) performance expectancy (3 items), (5) impact on image (2 items), (6) personal innovativeness (3 items), (7) acceptance (3 items), and (8) behavioral intention to use the technology (3 items). The items measured the constructs by asking participants to agree or disagree with statements using 5-point Likert-type scales, ranging from *strongly disagree* (1) to *strongly agree* (5). An item of the *image* construct (ie, Query 34, Image Expectancy 1; *People in my organization who use the tool have a high profile*), as well as the constructs *job security* and *facilitating conditions,* were dropped as they were irrelevant in the context under study. The final survey is depicted in the *Results* section. According to the original UTAUT model, gender, age, experience, and voluntariness of use are identified as moderators that affect technology beliefs and use [20]. Among these, we expected age to influence the attitude toward the use of the app, knowing that young adults have been more exposed to new technologies during their education and daily practice than older adults. Therefore, we selected age as a factor of interest to correlate with the technology acceptance self-administered survey items. Older participants were expected to have a greater reluctance in introducing the app into their daily practice.

Finally, a question using a 10-point Likert scale was administered to participants to measure their perceived satisfaction with the use of either the app or conventional preparation methods to prepare the drugs during the resuscitation scenarios (*on a scale of 1 to 10, how satisfied were you with your preparation experience?*). Data collection was conducted on site using Microsoft Excel spreadsheet (version 2011; Microsoft Corporation) and REDCap (Research Electronic Data Capture; Vanderbilt University) database. The investigators double-checked that the questionnaires were fully and accurately completed on site. Only study investigators had access to the data.

### Statistical Analysis

Age and work experience were assessed using the Spearman correlation coefficient. The mean global SUS score was reported using SD and 2-sided 95% CIs. Items on the SUS questionnaire were described using frequencies. For the technology acceptance self-administered survey questionnaire, scores on the 8 technology acceptance dimensions were described using the means, SDs, and frequencies of participants with a score of ≥4. The items were described in a similar manner. The association between dimension scores and participant ages was examined using the Spearman correlation coefficient. Paramedics and nurses were analyzed independently, without any comparison made. Statistical tests on the correlation coefficients were 2-tailed, with a significance level of 5%. Data analysis was conducted using R for Windows (version 4.0.2; R Core Team).

### Ethics Approval

Both trials received a declaration of no objection by the Geneva Cantonal Ethics Committee and Swiss Ethics as their purpose was to examine the effect of the intervention on health care providers. Both trials were registered at ClinicalTrials.gov (NCT03021122 and NCT03921346). The trials were conducted in accordance with the principles of the Declaration of Helsinki [51], Good Clinical Practice guidelines [52], the CONSORT-EHEALTH (Consolidated Standards of Reporting Trials of Electronic and Mobile Health Applications and Online TeleHealth; Multimedia Appendix 3) [39], and the Reporting Guidelines for Health Care Simulation Research [38].

## Results

### Overview

A total of 202 participants using the app (74, 36.6%, paramedics and 128, 63.4%, nurses) completed the scenarios and questionnaires without dropouts. Table 1 summarizes their demographic and health care characteristics.

**Table 1:** Baseline characteristics (N=202).

Characteristics			Paramedics (n=74)		Nurses (n=128)
Age (years), mean (SD)			35.7 (7.3)		37.2 (9.7)
**Age (years), n (%)**					
	<30	17 (23)		36 (28.1)	
	30-39	35 (47.3)		41 (32)	
	40-49	18 (24.3)		35 (27.3)	
	≥50	4 (5.4)		16 (12.5)	
**Gender, n (%)**					
	Female	25 (33.8)		121 (94.5)	
	Male	49 (66.2)		7 (5.5)	
Work experience in years since certification, mean (SD)			7.8 (6)		13.6 (9.1)
**Work experience in years since certification, n (%)**					
	<5	26 (35.1)		23 (18)	
	5-9	26 (35.1)		24 (18.8)	
	10-19	15 (20.3)		47 (36.7)	
	≥20	7 (9.5)		34 (26.6)	
**Smartphone user, n (%)**					
	No	0 (0)		5 (3.9)	
	Yes	74 (100)		123 (96.1)	
**Comfortable using a smartphone, n (%)**					
	Strongly disagree	1 (1.4)		14 (12.1)	
	Disagree	3 (4.1)		32 (27.6)	
	Neither disagree nor agree	12 (16.2)		55 (47.4)	
	Agree	38 (51.4)		15 (12.9)	
	Strongly agree	20 (27)		0 (0)	
	Missing data	0 (0)		12 (9.4)	
**Last preparation of emergency drugs (months)^a^, n (%)**					
	Never	9 (12.2)		57 (44.5)	
	<6	15 (20.3)		10 (7.8)	
	6-12	17 (23)		13 (10.2)	
	12-24	15 (20.3)		12 (9.4)	
	>20	18 (24.3)		36 (28.1)	
**Satisfaction with the media currently available to prepare emergency drugs, n (%)**					
	Strongly disagree	9 (12.2)		9 (7.1)	
	Disagree	18 (24.3)		23 (18.3)	
	Neither disagree nor agree	25 (33.8)		63 (50)	
	Agree	21 (28.4)		26 (20.6)	
	Strongly agree	1 (1.4)		5 (4)	
	Missing data	0 (0)		0 (0)	
**Mastering the preparation of emergency drugs, n (%)**					
	Strongly disagree	9 (12.2)		35 (27.3)	
	Disagree	15 (20.3)		37 (28.9)	
	Neither disagree nor agree	30 (40.5)		35 (27.3)	
	Agree	19 (25.7)		17 (13.3)	
	Strongly agree	1 (1.4)		4 (3.1)	
	Missing data	0 (0)		0 (0)	
**In favor of introducing technological tools to assist in emergency drug preparation, n (%)**					
	Strongly disagree	0 (0)		1 (0.8)	
	Disagree	0 (0)		0 (0)	
	Neither disagree nor agree	6 (8.1)		4 (3.1)	
	Agree	24 (32.4)		45 (35.2)	
	Strongly agree	44 (59.5)		78 (60.9)	
	Missing data	0 (0)		0 (0)	

^a^For the nurses, emergency drugs meant vasoactive drugs in continuous infusion.

Although there was a wide age distribution, most respondents were aged between 30 and 49 years, with the nursing group having slightly more older participants. A strong correlation (*r*=0.72; *P*<.001) between paramedics’ age and work experience, expressed in years, was found, as well as a very strong correlation (*r*=0.95; *P*<.001) for nurses.

### Usability Testing

**Figure 1:**
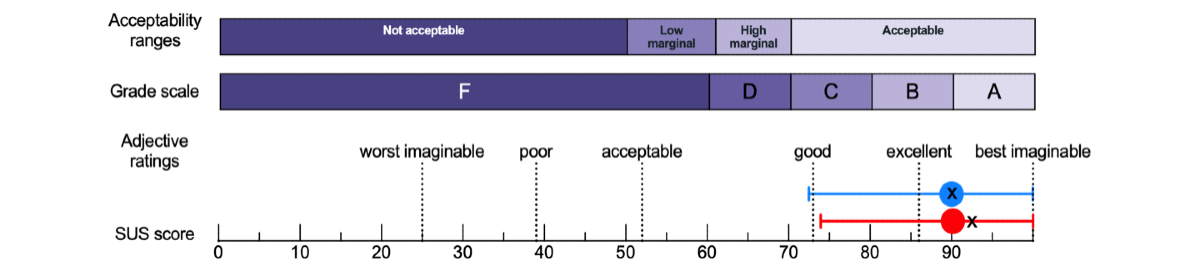
Overall System Usability Scale (SUS) scores to assess the usability of the PedAMINES (Pediatric Accurate Medication in Emergency Situations) app. The SUS score is located on a normalized scale ranging from a minimum score of 0 to a maximum of 100 [27]. Adjective ratings provide an interpretation of the SUS score [53]. The SUS also provides letter grades, similar to those used in the traditional school grading system [54]. The acceptability ranges indicate whether the tool is acceptable or not. Red dots represent the mean SUS score in paramedics and blue dots in nurses. Capped blue and red lines represent the 5th and 95th percentiles. Crosses represent medians (paramedics: 92.5, 5th-95th percentiles: 74.125-100; nurses: 90, 5th-95th percentiles: 72.5-100).

**Figure 2:**
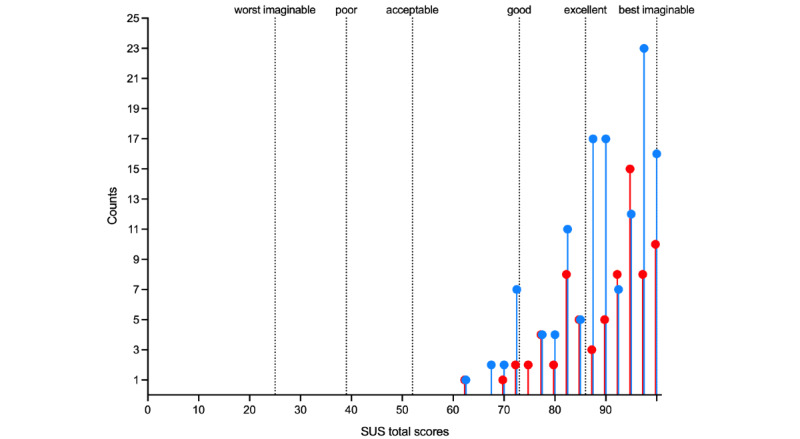
Distribution of counts of System Usability Scale (SUS) total scores. Red dots denote paramedics; blue dots denote nurses.

**Figure 3:**
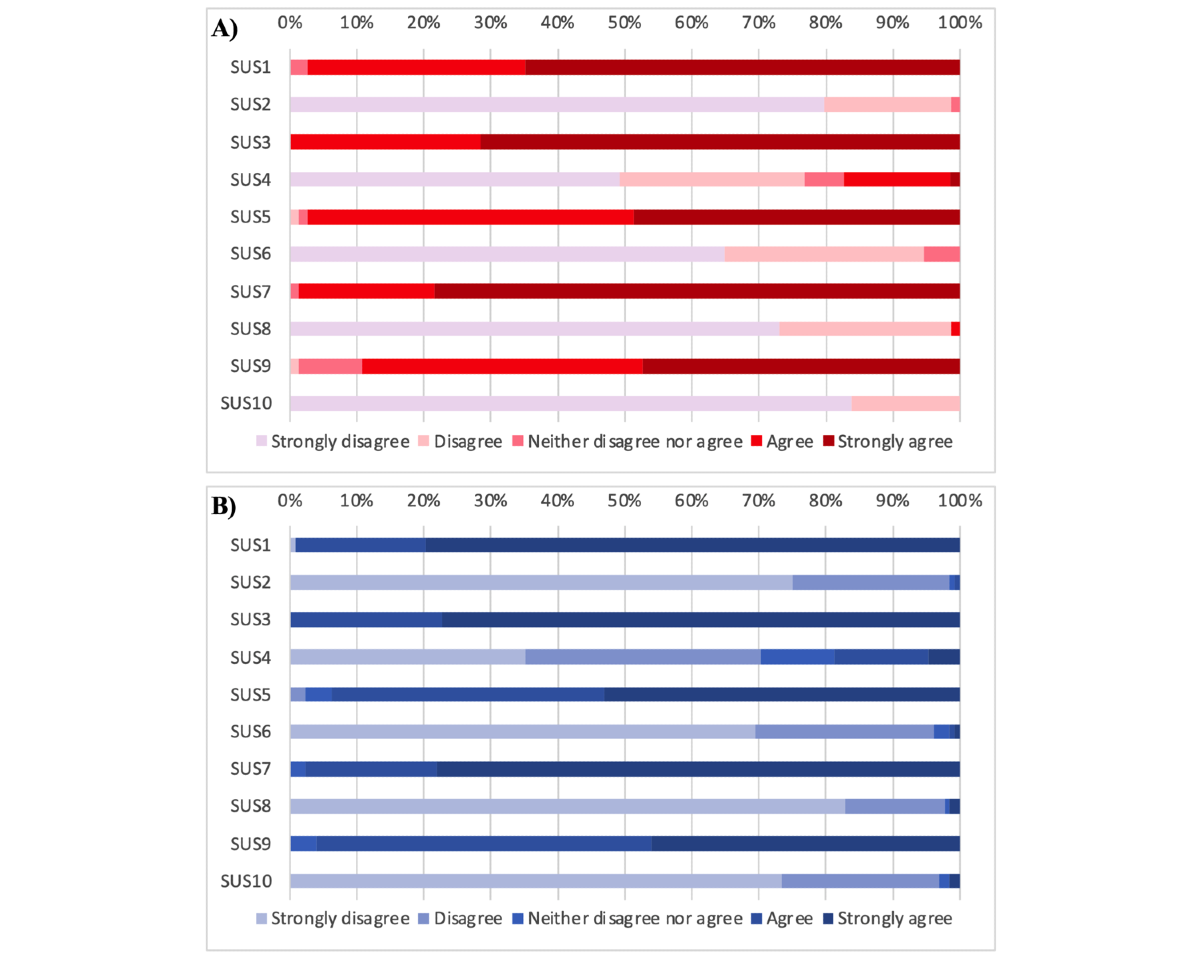
Percent distribution of item responses by (A) paramedics (n=74) and (B) nurses (n=128) on the (inversed) System Usability Scale (SUS) items. The SUS comprises 10 items (numbered as SUS1 to SUS10).

As shown in Figure 1, the mean total SUS scores for the app were 89.7 (SD 8.7; 95% CI 87.7-91.7) for paramedics and 89.5 (SD 8.8; 95% CI 88.0-91.1) for nurses’ quotation, which qualifies the tool as between *excellent* and the *best imaginable*, according to Bangor et al [53]. All scores were at least >60, spanning from 62.5 (2/202, 1% of people) to 100 (26/202, 12.9% of people; Figure 2). Figure 3 provides a visual overview of the distribution of the item responses on the SUS. SUS total score was not significantly associated with participants’ age (paramedics: *r*=−0.05 and *P*=.66; nurses: *r*=−0.01 and *P*=.91), which is likely related to highly skewed scores toward high usability with little variability among respondents (Multimedia Appendix 4). The other demographic moderators detailed in Table 1 did not have any significant effect on participants’ acceptance of the app.

### Technology Acceptance

Table 2 shows the overall perceptions of paramedics and nurses regarding the mobile app according to the adapted technology acceptance self-administered survey constructs. The paramedics and nurses largely agreed (mostly with scores ≥4) that the app (1) could enhance their performance during emergency drug preparation (perceived usefulness), (2) was easy to use (perceived ease of use), (3) met the requirements necessary to effortlessly address the complexity of the drug preparation task when handled by a technology tool (TTF), (4) could help them achieve performance gains during this procedure (performance expectancy), (5) elicited their intention to use it (attitude toward using a technology), (6) is acceptable for emergency drug preparation (acceptance), and (7) would be intentionally used for this purpose over a long period (intention to use). Intention to use the app was the highest-rated construct, with the item *Assuming I had access to PedAMINES, I intend to use it* receiving the highest agreement among participants (Table 2). The paramedics and nurses agreed to a lesser extent that the app would enhance the adopter’s social image or status in their organization. Participants had the least agreement on the item *Having PedAMINES will be a status symbol in my organization* from the image construct. As with the SUS, the technology acceptance self-administered survey scores were skewed toward high scores of acceptance of the app and intention to use it. Only the constructs *image, attitude toward technology use,* and *acceptance* showed a somewhat greater spread across the score range. A weak but significantly negative correlation between nurses’ age and attitude toward app use (r=−0.27; *P*=.003) was identified. Although the TAM used here or the original UTAUT from which it was derived can be influenced by four key moderating variables (ie, age, experience, gender, and voluntariness of use), the effect of the latter two on the acceptance constructs was not analyzed in this study, given the lack of significant variation in these moderators across individuals in the same setting [55]. Experience was highly correlated with age and, therefore, was not analyzed. No significant correlation between age and other items of the technology acceptance self-administered survey constructs was found, and no correlation was observed among paramedics. Multimedia Appendix 5 presents the results of the technology acceptance self-administered survey items by score range.

### Satisfaction

The app obtained a mean overall satisfaction score of 9.1 (SD 0.9) out of 10 among paramedics and 9.4 (SD 1.0) among nurses.

**Table 2:** Results of the technology acceptance survey by items.

Constructs and items			Definition^a^ and item wording		Paramedics (n=74)					Nurses (n=128)		
					Values, mean^b^ (SD)		Score ≥4, n (%)		Values, mean (SD)			Score ≥4, n (%)
**PU^c^**			The degree to which an individual perceives that using the system leads to enhanced personal performance [15,18]		4.69 (0.44)		66 (89.1)		4.79 (0.37)			122 (95.3)
	PU1	Using PedAMINES^d^ helps me to prepare emergency drugs more quickly.		4.69 (0.62)		68 (91.9)		4.88 (0.32)			128 (100)	
	PU2	Using PedAMINES helps me to prepare emergency drugs better.		4.73 (0.58)		71 (95.9)		4.74 (0.52)			123 (96.1)	
	PU3	Using PedAMINES makes it easier for me to prepare emergency drugs.		4.69 (0.52)		72 (97.3)		4.81 (0.51)			126 (98.4)	
	PU4	Using PedAMINES enhances my effectiveness in drug preparation.		4.66 (0.50)		73 (99)		4.74 (0.61)			121 (94.5)	
**PEOU^e^**			The degree to which an individual perceives that using the system will be free from physical or mental efforts [15,18,56]		4.61 (0.35)		72 (97.3)		4.76 (0.28)			126 (98.4)
	PEOU1	It is easy to get PedAMINES to do what l want it to do.		4.41 (0.94)		63 (85.1)		4.78 (0.47)			125 (97.7)	
	PEOU2	Overall, I find PedAMINES is easy to use.		4.74 (0.47)		73 (98.6)		4.86 (0.35)			128 (100)	
	PEOU3	It is easy for me to become skillful in using PedAMINES.		4.89 (0.31)		74 (100)		4.88 (0.33)			128 (100)	
	PEOU4	I often become confused with PedAMINES’ features when I used it.		4.41 (0.55)		72 (97.3)		4.53 (0.59)			124 (96.9)	
**TTF^f,g^**			The degree to which an individual perceives that using the system fits the requirements of a particular task [17,44,48]		4.49 (0.54)		63 (85.1)		4.64 (0.43)			121 (95.3)
	TTF1	PedAMINES has the functionalities l need to accomplish my tasks.		4.55 (0.62)		71 (95.9)		4.69 (0.52)			126 (98.4)	
	TTF2	PedAMINES’ functionalities give me exactly what I need for my work.		4.47 (0.67)		67 (91)		4.61 (0.54)			125 (97.7)	
	TTF3	PedAMINES is very well suited to my work.		4.70 (0.54)		71 (95.9)		4.77 (0.44)			127 (99.2)	
	TTF4	Using PedAMINES is compatible with most aspects of my work.		4.22 (0.90)		65 (87.8)		4.50 (0.68)			116 (91.3)	
**PE^h^**			The degree to which an individual perceives that using the system will help the user attain gains in job performance [20,44]		4.55 (0.59)		61 (82.4)		4.58 (0.57)			115 (89.8)
	PE1	Using PedAMINES, I get better chances to improve my professional position.		4.28 (0.96)		59 (79.7)		4.21 (1.0)			101 (78.9)	
	PE2	Using PedAMINES will help me improve or continue to help to improve emergency drugs preparation.		4.61 (0.74)		67 (90.5)		4.77 (0.55)			125 (97.7)	
	PE3	Using PedAMINES will increase the quality of my drug preparation.		4.74 (0.57)		71 (95.9)		4.77 (0.52)			124 (96.9)	
**II^i,j^**			The extent to which an individual perceives that using the system enhances one’s image or status in ones’ social system [20,50]		3.46 (0.96)		30 (40.5)		3.24 (0.98)			41 (32.5)^k^
	II1	People in my practice setting who use PedAMINES will have more prestige than those who do not.		3.84 (1.06)		47 (63.5)		3.53 (1.13)			71 (55.9)^l^	
	II2	Using PedAMINES will be a status symbol in my practice setting.		3.08 (1.16)		22 (29.7)		2.94 (1.09)			36 (28.6)^k^	
**PI^m,n^**			The extent to which an individual has an innate propensity (willingness) toward trying any new technology [50,57]		4.14 (0.58)		48 (64.9)		3.97 (0.77)			73 (57.5)^l^
	PI1	If I heard about a new technology, I would look for ways to experiment with it.		4.55 (0.58)		71 (95.9)		4.39 (0.73)			116 (91.3)^l^	
	PI2	Among my peers, I am usually the first to try out new technologies.		3.59 (0.95)		42 (56.8)		3.42 (1.03)			62 (48.8)^l^	
	PI3	I like to experiment with new technologies.		4.28 (0.67)		65 (87.8)		4.09 (0.85)			102 (80.3)^l^	
**A^o,p^**			The extent to which individuals accept to use a new technology [56]		4.32 (0.61)		56 (75.7)		4.30 (0.63)			100 (79.4)^k^
	A1	In my opinion, it would be desirable to use PedAMINES in addition to conventional preparation methods for emergency drugs.		4.53 (0.74)		69 (93.2)		4.40 (0.90)			111 (88.1)^k^	
	A2	It would be good to use PedAMINES more than the conventional methods for the preparation of emergency drugs.		4.39 (0.70)		67 (90.5)		4.45 (0.78)			114 (89.8)^l^	
	A3	I think it would be highly desirable to use only PedAMINES instead of conventional methods for the preparation of emergency drugs.		4.04 (1.07)		54 (73)		4.06 (1.14)			94 (74)^l^	
**BI^q,r^**			Individuals’ subjective intention toward using a technology over a longer period [21]		4.81 (0.34)		72 (97.3)		4.82 (0.39)			125 (98.4)^l^
	BI1	Assuming I had access to PedAMINES, I intend to use it.		4.68 (0.60)		69 (93.2)		4.81 (0.41)			126 (99.2)^l^	
	BI2	I predict I would use PedAMINES in the next 6 months.		4.91 (0.29)		74 (100)		4.85 (0.42)			126 (99.2)^l^	
	BI3	I expect my use of PedAMINES to continue in the future.		4.84 (0.37)		74 (100)		4.80 (0.44)			125 (98.4)^l^	

^a^Presented with the source references from which the items were derived and adapted for the context of this study.

^b^Each item was measured on a 5-point Likert-type scale (1=*strongly disagree* and 5=*strongly agree*); the higher the score, the more agreement with the statement.

^c^PU: perceived usefulness.

^d^PedAMINES: Pediatric Accurate Medication in Emergency Situations.

^e^PEOU: perceived ease of use.

^f^TTF: task–technology fit.

^g^Missing data from nurses, n=1.

^h^PE: performance expectancy.

^i^II: image.

^j^Missing data under nurses, n=2.

^k^N=126.

^l^N=127.

^m^PI: personal innovativeness.

^n^Missing data under nurses, n=1.

^o^A: acceptance.

^p^Missing data under nurses, n=2.

^q^BI: behavioral intention to use.

^r^Missing data under nurses, n=1.

## Discussion

### Principal Findings

The main finding of our study was that the usability assessment of the PedAMINES app scored high on all items of the SUS questionnaire, with >86% of paramedics and >93% of nurses rating the overall usability of the app as excellent (ie, scoring >80 on the SUS). This suggests that the app is highly usable by users targeted for its purpose and appears to be an accepted supportive tool. This usability observed regardless of years of experience, age, or gender suggests a worthwhile benefit of its use by novice emergency care providers and those with limited exposure to children who are critically ill and few opportunities to prepare emergency medications in pediatric doses, particularly in general hospitals handling pediatric emergencies and in the prehospital setting. Leveling providers’ compounding skills could indeed prove to be an advantage in high-stakes clinical events with infrequent occurrences, such as pediatric cardiopulmonary resuscitation. To our knowledge, this is the first study to report usability testing and the intent of paramedics and nurses to use a mobile app as a supportive digital tool for emergency drug preparation in pediatric life-threatening situations. The fact that participants who were previously naive about the PedAMINES app were able to use it correctly to significantly reduce medication error rates [32,33] after a single 5-minute prescenario training and broadly agree on its usability validated the user-centered design [58-60], the Fitts laws [61], and progressive disclosure [62] principles that underpinned its iterative development process [63]. This approach has proven beneficial in allowing end users to influence the development process and increase the final usability of web-based HIT [64,65]. To provide some perspective, the mean total SUS scores >89 in this study were higher than those reported in a comparative study of the top 10 nonmedical mobile apps evaluated by >3500 users [66]. The SUS has proven to be a highly robust and versatile tool for collecting users’ subjective evaluations of a product’s usability [67]. In high-stakes, critical situations where time is of the essence, the usability of dedicated apps must be high as there is no room at that moment to become familiar with the app [68]. This study suggests that endowing paramedics and nurses with usability-proven mobile apps might contribute to improving the safety of the drug administration process in pediatric emergency care.

To limit and mitigate the likelihood of medication errors, several assistive eHealth technologies have been developed over the past decades to target and support individual medication steps [34]. However, before an eHealth tool such as a mobile app can be adopted and implemented into clinical practice to support the delivery of health care, usability testing and the likelihood that it will be accepted as an aid by its future users are prerequisites for success [69,70]. Usability (ie, the extent to which a system, product, or service can be used by end users to achieve specific goals with effectiveness, efficiency, and satisfaction in a given context of use [71]) is recognized as a key quality factor in determining the success and adoption of an app [26]. Usability testing of an app with end users early in the process may also uncover potential issues related to poor app development and design, which could otherwise ultimately lead to endangering patient safety [72,73]. Although previous research has shown that mHealth apps can help improve the quality and safety of care [74], functional characteristics have often been privileged to the detriment of the needs and characteristics of end users [23]. Consequently, users may be reluctant to adopt them or only use them for a short span of time after their introduction and then abandon them [75,76]. To fully anticipate their acceptance and long-term adoption rate, it is essential to look beyond the technology itself and its usability by also considering end users’ beliefs, perceptions, and intentions regarding its use [24].

To date, only a small fraction of mHealth apps have published their usability evaluation results, with most apps developed in the commercial sector that have rarely been published in the scientific literature [26]. In the field of cardiopulmonary resuscitation, a recent study identified 34 available mobile apps on Google Play and Apple App stores [68]. However, many of these apps are marginally medically correct, have not been validated in evidence-based studies, and have limited usability [77]. Of the few available medical mobile apps that offer weight-based drug dosing, such as Handtevy Mobile [78], SafeDose Mobile (eBroselow) [79], Infinite Dose PRO [80], Pedi QuikCalc [81], PEDeDose [82], and EZDrips Peds [83], none have shown proven results of their efficacy, especially in terms of usability.

Although studies have been conducted in recent years to assess user attitudes toward new HIT, there is a lack of research on the perception and acceptance of mHealth technologies by health care providers [84]. In this study, we evaluated 8 technology acceptance constructs to capture the different aspects that could drive the intention to use the PedAMINES app from the perspective of emergency care professionals. We report that paramedics and nurses had an overall acceptant attitude toward the app and agreed with its use as a means of supporting the drug preparation process during emergency care. Among the identified constructs that drive technology acceptance, participants strongly agreed with the usefulness, ease of use, TTF, performance, and behavioral intention to use the app, with mean total scores >4.5/5. In addition, attitudes toward use and acceptance were positive. As these constructs have been shown to be among the most important factors driving individuals to adopt mHealth apps [69,75,85,86], the results of our study provide support for the future adoption of our app by nurses and paramedics in emergency care. However, long-term adoption studies in clinical practice need to be conducted to confirm this. One of the strengths of the study is that the evaluation of the app did not focus on the behavioral intent of its adoption under *traditional* laboratory-based conditions but took into account the usual context of its use by evaluating its usability and acceptability in simulated, in situ conditions very close to the reality in which the app should ultimately be deployed [73].

Our study also found that nurses’ attitudes toward app use were slightly negatively influenced by age, whereas usability ratings were not. Consistent with previous research, this may be as some form of resistance may characterize the attitude toward the use of new technologies among nurses with longer experience and their reliance on their own prior learning without the use of technology to assist them [87-89]. Paramedics were represented in younger age categories than nurses, which may explain the lack of a significant age influence on their attitude toward the app. However, previous studies on the effect of age on attitudes toward novelty have yielded controversial results, and caution should be exercised about this interpretation related to the influence of age, and this aspect requires further study [87]. Of note, our study also found that images appeared to be the least relevant technology acceptance construct. Similar to a previous study [90], this could be explained by the nature of the intervention, as the impact of an app on the image of its users among their peers can presumably only be properly assessed through long-term use in routine practice and not through a single use, as was the case in this study. Future research should determine whether the acceptability of the app, as assessed through this simulation-based study, will translate into its long-term use in real-life situations, as the success of an app likely depends primarily on sustained use over time rather than on its first use [91,92]. Meanwhile, this study generated useful knowledge to guide future developments of other mHealth interventions using in situ high-fidelity simulation and the SUS and technology acceptance self-administered survey questionnaires as a basis for exploring the usability and acceptability of mobile apps at the point of care in emergency medicine.

### Limitations

This study has some limitations. First, the app was evaluated in simulation-based trials, and its usability and acceptance in real-life situations might be different. However, the simulated scenarios allowed for consistent assessment of the app among participants in a standardized manner, which would have been difficult to achieve in actual cardiopulmonary resuscitations. In situ clinical simulation, which involves observing representative users performing tasks in their representative environments, has proven to be a valuable way of addressing specific factors that influence technology usability and adoption and ensuring that the results obtained can be generalized to the real world [73]. Second, the survey used to assess the acceptance of the app was not the original UTAUT model but an adapted version with additional valid and reliable constructs. Adding constructs to the original framework is a common practice [93]. As suggested by Venkatesh et al [21], the addition of relevant constructs to the original UTAUT model, where each construct can be interpreted independently of the others, may contribute to extending its applicability to other contexts in an attempt to better understand factors influencing adoption and behavioral intention to use a technology. For example, the TTF construct was used here to evaluate the users’ perception of the complexity of the task to be handled by being supported by the app. Third, this study was limited to the evaluation of the app in the context of drug preparations at pediatric doses. It does not provide information on its usability and acceptance if it were to be used to support the preparation of drugs for adults. Fourth, no qualitative usability testing was conducted in the field as part of this study so as to not detract from the scenario and primary outcome. More extensive qualitative laboratory and field testing of the app will be the focus of future research. Finally, the SUS questionnaire measured users’ perceived usability of the app rather than its actual usability. Users were not informed of their performance after the scenarios and only filled out the questionnaire based on their perception of their app use. Future studies must consider assessing objective PedAMINES usability metrics, such as task completion rate and efficiency on time on task.

### Conclusions

This study evaluated the usability and technology acceptance of a mobile app to assist in the preparation of emergency drugs at the point of care by skilled nurses and advanced paramedics during simulated pediatric cardiopulmonary resuscitations. We found excellent usability and high technology acceptance. This provides information not only about the initial adoption of the app by these caregivers but also, more broadly, about the likelihood of successful adoption of mHealth apps in emergency care that have previously gone through such a usability and technology acceptance evaluation process. Our findings contribute to the exploration of factors influencing the usability and acceptance of mHealth apps by emergency caregivers, particularly when devolved to pediatrics, and lay the groundwork for future clinical practice and long-term research.

## Appendix

Multimedia Appendix 1 The System Usability Scale questionnaire.

Multimedia Appendix 2 The technology acceptance survey for the evaluation of health professionals’ acceptance of a mobile app (PedAMINES [Pediatric Accurate Medication in Emergency Situations]) for pediatric drug preparation.

Multimedia Appendix 3 CONSORT-EHEALTH (Consolidated Standards of Reporting Trials of Electronic and Mobile Health Applications and Online TeleHealth) checklist (version 1.6.1).

Multimedia Appendix 4 Results of the System Usability Scale items for all participants by occupation type (paramedics or nurses).

Multimedia Appendix 5 Results of the technology acceptance survey items by occupation type (paramedics or nurses) and score ranges.

## Abbreviations

CONSORT: Consolidated Standards of Reporting Trials
CONSORT-EHEALTH: Consolidated Standards of Reporting Trials of Electronic and Mobile Health Applications and Online TeleHealth
EMS: emergency medical service
HIT: health information technology
mHealth: mobile health
OHCA: out-of-hospital cardiac arrest
PedAMINES: Pediatric Accurate Medication in Emergency Situations
REDCap: Research Electronic Data Capture
RN: registered nurse
SUS: System Usability Scale
TAM: Technology Acceptance Model
TTF: task–technology fit
UTAUT: Unified Theory of Acceptance and Use of Technology
