# Supplementary material for: Usability Testing and Technology Acceptance of an mHealth App at the Point of Care During Simulated Pediatric In- and Out-of-Hospital Cardiopulmonary Resuscitations: Study Nested Within 2 Multicenter Randomized Controlled Trials
Source: JMIR Hum Factors. 2022 Mar 1;9(1):e35399. doi: 10.2196/35399 (PMC8924787; doi:10.2196/35399)
Supplement: Multimedia Appendix 1 [file humanfactors_v9i1e35399_app1.docx]

**Multimedia appendix 1.** The System Usability Scale (SUS) questionnaire

| (1) I think that I would like to use this system frequently. |
| --- |
| (2) I found the system unnecessarily complex. |
| (3) I thought the system was easy to use. |
| (4) I think that I would need the support of a technical person to be able to use this system. |
| (5) I found the various functions in this system were well integrated. |
| (6) I thought there was too much inconsistency in this system. |
| (7) I would imagine that most people would learn to use this system very quickly. |
| (8) I found the system very cumbersome to use. |
| (9) I felt very confident using the system. |
| (10) I needed to learn a lot of things before I could get going with this system. |

The SUS consists of a 10-item questionnaire with five response options for each item based on their level of agreement and ranging from 1 (‘strongly disagree’) to 5 (‘strongly agree’). Adapted from [1].

To obtain a score, follow these steps:

1. Odd numbered (1,3,5,7,9) statements (the positively worded items) = score – 1
2. Even numbered (2,4,6,8,10) statements (the negatively worded items) = score – 5
3. Total SUS = add the scores of each item and multiply by 2.5

[1] Brooke J. SUS: a quick and dirty usability scale: London: Taylor and Francis; 1996.
