# Supplementary material for: Usability Testing and Technology Acceptance of an mHealth App at the Point of Care During Simulated Pediatric In- and Out-of-Hospital Cardiopulmonary Resuscitations: Study Nested Within 2 Multicenter Randomized Controlled Trials
Source: JMIR Hum Factors. 2022 Mar 1;9(1):e35399. doi: 10.2196/35399 (PMC8924787; doi:10.2196/35399)
Supplement: Multimedia Appendix 2 [file humanfactors_v9i1e35399_app2.docx]

**Multimedia appendix 2.** The Technology Acceptance Survey for the evaluation of health professional’s acceptance of a mobile app (PedAMINES) for pediatric drug preparation.

1. Read the 26 statements of the questionnaire presented.
2. Rate each statement using the scale proposed by checking the box that best match your choice.

| **1**  **Strongly disagree** | **2**  **Disagree** | **3**  **Neither agree nor disagree** | **4**  **Agree** | **5**  **Strongly agree** |
| --- | --- | --- | --- | --- |

| 1. Using PedAMINES helps me to prepare emergency drugs more quickly | 1 | 2 | 3 | 4 | 5 |
| --- | --- | --- | --- | --- | --- |
| 1. Using PedAMINES helps me to prepare emergency drugs better | 1 | 2 | 3 | 4 | 5 |
| 1. Using PedAMINES makes it easier for me to prepare emergency drugs | 1 | 2 | 3 | 4 | 5 |
| 1. Using PedAMINES enhances my effectiveness in drug preparation | 1 | 2 | 3 | 4 | 5 |
| 1. It is easy to get PedAMINES to do what l want it to do | 1 | 2 | 3 | 4 | 5 |
| 1. Overall, I find PedAMINES is easy to use | 1 | 2 | 3 | 4 | 5 |
| 1. It is easy for me to become skillful in using PedAMINES | 1 | 2 | 3 | 4 | 5 |
| 1. I often become confused with PedAMINES’ features when I used it | 1 | 2 | 3 | 4 | 5 |
| 1. PedAMINES has the functionalities l need to accomplish my tasks | 1 | 2 | 3 | 4 | 5 |
| 1. PedAMINES’ functionalities give me exactly what I need for my work | 1 | 2 | 3 | 4 | 5 |
| 1. PedAMINES is very well suited to my work | 1 | 2 | 3 | 4 | 5 |
| 1. Using PedAMINES is compatible with most aspects of my work | 1 | 2 | 3 | 4 | 5 |
| 1. Using PedAMINES, I get better chances to improve my professional position | 1 | 2 | 3 | 4 | 5 |
| 1. Using PedAMINES will help me improve or continue to help to improve emergency drugs preparation | 1 | 2 | 3 | 4 | 5 |
| 1. Using PedAMINES will increase the quality of my drug preparations | 1 | 2 | 3 | 4 | 5 |
| 1. People in my practice setting who use PedAMINES will have more prestige than those who do not | 1 | 2 | 3 | 4 | 5 |
| 1. Using PedAMINES will be a status symbol in my practice setting | 1 | 2 | 3 | 4 | 5 |
| 1. If I heard about a new technology, I would look for ways to experiment with it | 1 | 2 | 3 | 4 | 5 |
| 1. Among my peers, I am usually the first to try out new technologies | 1 | 2 | 3 | 4 | 5 |
| 1. I like to experiment with new technologies | 1 | 2 | 3 | 4 | 5 |
| 1. In my opinion, it would be desirable to use PedAMINES in addition to conventional preparation methods for emergency drugs | 1 | 2 | 3 | 4 | 5 |
| 1. It would be good to use PedAMINES more than the conventional methods for the preparation of emergency drugs | 1 | 2 | 3 | 4 | 5 |
| 1. I think it would be highly desirable to use only PedAMINES instead of conventional methods for the preparation of emergency drugs | 1 | 2 | 3 | 4 | 5 |
| 1. Assuming I had access to PedAMINES, I intend to use it | 1 | 2 | 3 | 4 | 5 |
| 1. I predict I would use PedAMINES in the next 6 months | 1 | 2 | 3 | 4 | 5 |
| 1. I expect my use of PedAMINES to continue in the future | 1 | 2 | 3 | 4 | 5 |

Adapted from Ibanescu G. Acceptance factors and use of information technologies: an empirical study on the use of "Rational Suite" software by the employees of a large IT services company: Quebec University (UQAM). Montreal (Quebec); 2011.
