# Supplementary material for: Usability Testing and Technology Acceptance of an mHealth App at the Point of Care During Simulated Pediatric In- and Out-of-Hospital Cardiopulmonary Resuscitations: Study Nested Within 2 Multicenter Randomized Controlled Trials
Source: JMIR Hum Factors. 2022 Mar 1;9(1):e35399. doi: 10.2196/35399 (PMC8924787; doi:10.2196/35399)
Supplement: Multimedia Appendix 4 [file humanfactors_v9i1e35399_app4.docx]

**Multimedia appendix 4.** Results of the System Usability Scale (SUS) items for all participants by occupation type (paramedics or nurses).

|  | **Values per 5-point Likert scale response^a^, n (%)** | | | | | | | | | | | |
| --- | --- | --- | --- | --- | --- | --- | --- | --- | --- | --- | --- | --- |
|  | **Paramedics (N=74)** | | | | |  | | **Nurses (N=128)** | | | | |
|  | 1 | 2 | 3 | 4 | 5 |  | | 1 | 2 | 3 | 4 | 5 |
| 1. I think that I would like to use PedAMINES frequently. | 0  (0) | 0  (0) | 2  (2.7) | 24  (32.4) | 48  (64.9) |  | 1  (0.8) | | 0  (0) | 0  (0) | 25  (19.5) | 102  (79.7) |
| 2. I found PedAMINES unnecessarily complex. | 59  (79.7) | 14  (18.9) | 1  (1.4) | 0  (0) | 0  (0) |  | 96  (75) | | 30  (23.4) | 1  (0.8) | 1  (0.8) | 0  (0) |
| 3. I thought PedAMINES was easy to use. | 0  (0) | 0  (0) | 0  (0) | 21  (28.4) | 53  (71.6) |  | 0  (0) | | 0  (0) | 0  (0) | 29  (22.7) | 99  (77.3) |
| 4. I think that I would need the support of a technical person to be able to use PedAMINES. | 34  (45.9) | 24  (32.4) | 4  (5.4) | 11  (14.9) | 1  (1.4) |  | 45  (35.2) | | 45  (35.2) | 14  (10.9) | 18  (14.1) | 6  (4.7) |
| 5. I found the various functions in PedAMINES were well integrated. | 0  (0) | 1  (1.4) | 1  (1.4) | 36  (48.6) | 36  (48.6) |  | 0  (0) | | 3  (2.3) | 5  (3.9) | 52  (40.6) | 68  (53.1) |
| 6. I thought there was too much inconsistency in PedAMINES. | 48  (64.9) | 22  (29.7) | 4  (5.4) | 0  (0) | 0  (0) |  | 89  (69.5) | | 34  (26.6) | 3  (2.3) | 1  (0.8) | 1  (0.8) |
| 7. I would imagine that most people would learn to use PedAMINES very quickly. | 0  (0) | 0  (0) | 1  (1.4) | 15  (20.3) | 58  (78.4) |  | 0  (0) | | 0  (0) | 3  (2.3) | 25  (19.5) | 100  (78.1) |
| 8. I found PedAMINES very cumbersome to use. | 54  (73) | 19  (25.7) | 0  (0) | 1  (1.4) | 0  (0) |  | 106  (82.8) | | 19  (14.8) | 1  (0.8) | 0  (0) | 2  (1.6) |
| 9. I felt very confident using PedAMINES. | 0  (0) | 1  (1.4) | 7  (9.5) | 31  (41.9) | 35  (47.3) |  | 0  (0) | | 0  (0) | 5  (3.9) | 64  (50) | 59  (46.1) |
| 10 I needed to learn a lot of things before I could get going with PedAMINES. | 62  (83.8) | 12  (16.2) | 0  (0) | 0  (0) | 0  (0) |  | 95  (74.2) | | 30  (23.4) | 2  (1.6) | 0  (0) | 1  (0.8) |
| ^a^ From 1 ‘strongly disagree’ to 5 ‘strongly agree’. | | | | | | | | | | | | |
