# Supplementary material for: Usability Testing and Technology Acceptance of an mHealth App at the Point of Care During Simulated Pediatric In- and Out-of-Hospital Cardiopulmonary Resuscitations: Study Nested Within 2 Multicenter Randomized Controlled Trials
Source: JMIR Hum Factors. 2022 Mar 1;9(1):e35399. doi: 10.2196/35399 (PMC8924787; doi:10.2196/35399)
Supplement: Multimedia Appendix 5 [file humanfactors_v9i1e35399_app5.docx]

**Multimedia appendix 5.** Results of the Technology Acceptance Survey (TAS) items by occupation type (paramedics or nurses) and score ranges.

|  | **Values per 5-point Likert scale response^a^, per score ranges, n (%)** | | | | |
| --- | --- | --- | --- | --- | --- |
| **Technology Acceptance items** | 1 to 1.99 | 2 to 2.99 | 3 to 3.99 | 4 to 4.99 | 5 |
| **Paramedics (N=74)** |  |  |  |  |  |
| PU | 0 (0.0) | 0 (0.0) | 8 (10.8) | 25 (33.8) | 41 (55.4) |
| PEU | 0 (0.0) | 0 (0.0) | 2 (2.7) | 55 (74.3) | 17 (23.0) |
| TTF | 0 (0.0) | 0 (0.0) | 11 (14.9) | 42 (56.8) | 21 (28.4) |
| PE | 0 (0.0) | 0 (0.0) | 13 (17.6) | 24 (32.4) | 37 (50.0) |
| I | 2 (2.7) | 13 (17.6) | 29 (39.2) | 20 (27.0) | 10 (13.5) |
| ATT | 0 (0.0) | 1 (1.4) | 25 (33.8) | 37 (50.0) | 11 (14.9) |
| A | 0 (0.0) | 0 (0.0) | 18 (24.3) | 33 (44.6) | 23 (31.1) |
| ITU | 0 (0.0) | 0 (0.0) | 2 (2.7) | 22 (29.7) | 50 (67.6) |
| **Nurses (N=128)** |  |  |  |  |  |
| PU | 0 (0.0) | 0 (0.0) | 6 (4.7) | 36 (28.1) | 86 (67.2) |
| PEU | 0 (0.0) | 0 (0.0) | 2 (1.6) | 69 (53.9) | 57 (44.5) |
| TTF | 0 (0.0) | 0 (0.0) | 6 (4.7) | 59 (46.5) | 62 (48.8) |
| PE | 0 (0.0) | 1 (0.8) | 12 (9.4) | 52 (40.6) | 63 (49.2) |
| I | 8 (6.3) | 25 (19.8) | 52 (41.3) | 33 (26.2) | 8 (6.3) |
| ATT | 1 (0.8) | 9 (7.1) | 44 (34.6) | 57 (44.9) | 16 (12.6) |
| A | 0 (0.0) | 2 (1.6) | 23 (18.4) | 66 (52.8) | 34 (27.2) |
| ITU | 0 (0.0) | 1 (0.8) | 1 (0.8) | 29 (22.8) | 96 (75.6) |

^a^ From 1 ‘strongly disagree’ to 5 ‘strongly agree’.

PU, perceived usefulness; PEU, perceived ease of use; TTF, task technology task fit; PE, performance expectancy; I, image; ATT, attitude towards using technology; A, acceptance; ITU, intention to use.
